# Granulometric characterization of airborne particulate release during spray application of nanoparticle-doped coatings

**DOI:** 10.1007/s11051-014-2520-1

**Published:** 2014-07-04

**Authors:** Daniel Göhler, Michael Stintz

**Affiliations:** Research Group Mechanical Process Engineering, Institute of Process Engineering and Environmental Technology, Technische Universität Dresden, Münchner Platz 3, 01062 Dresden, Germany

**Keywords:** Nanoparticle release, Airborne particle emission, Spray application, Spray can, Spray gun, Nanoparticle-doped coatings, Risk assessment

## Abstract

Airborne particle release during the spray application of coatings was analyzed in the nanometre and micrometre size range. In order to represent realistic conditions of domestic and handcraft use, the spray application was performed using two types of commercial propellant spray cans and a manual gravity spray gun. Four different types of coatings doped with three kinds of metal-oxide tracer nanoparticle additives (TNPA) were analyzed. Depending on the used coating and the kind of spray unit, particulate release numbers between 5 × 10^8^ and 3 × 10^10^ particles per gram ejection mass were determined in the dried spray aerosols. The nanoparticulate fraction amounted values between 10 and 60 no%. The comparison between nanoparticle-doped coatings with non-doped ones showed no TNPA-attributed differences in both the macroscopic spray process characteristics and the particle release numbers. SEM, TEM and EDX-analyzes showed that the spray aerosols were composed of particles made up solely from matrix material and sheathed pigments, fillers and TNPAs. Isolated ZnO- or Fe_2_O_3_-TNPAs could not be observed.

## Introduction

Nanomaterials are affected by the evolving risk discussions between stakeholders in research, governments, regulators, non-governmental organizations and industry, but currently no harmonized definition of the term nanomaterial exists under regulatory aspects.

A science-based terminology for nanomaterials is given in ISO/TS 80004-1:^2010^, wherein a distinction is drawn between nano-objects and nanostructured materials. The identifying feature of nanostructured materials is their internal or surface structure in the nanoscale (≤100 nm), but their external dimensions are typically greater. Beside the ISO terminology, the European Commission issued a recommendation on the definition of nanomaterials (2011/696/EU) (European Commission 2011). This definition comprises “natural, incidental or manufactured materials containing particles in an unbound state or as an aggregate or as an agglomerate and where, for 50 % or more of the particles in the number size distribution, one or more external dimension is in the size range 1–100 nm”. Consequently, the EC-definition based solely on the size of the constituent particles of the material, without regard to material properties or to hazard and risk.

The coating industry processes numerous materials within their products (e.g. dispersing agents, pigments, fillers), which are covered by both the recommendation of the European Commission and ISO/TS 80004-1:^2010^. In this context, a particulate risk assessment is reasonable for this sector of industry.

 Besides the hazard profile of a substance, the second fundamental premise to perform an overall risk assessment is the exposure (e.g. NRC 1983). Kuhlbusch et al. (2011) reviewed published studies on exposure characterization by workplace measurement and laboratory analyses and concluded that the latter ones can provide basic information about the ability and the quantity of airborne nanoparticle release. In this context, a recently published review (Froggett et al. 2014) pointed out that only the half of the 54 available release studies for solid nanocomposites have an experimental nature, whereas the others ones have a more observational character. Froggett et al. (2014) concluded that future work should spend more attention on the release processes itself. Nevertheless, Brouwer (2010) showed that several potential release scenarios for engineered nano-objects (ENO) exist in the whole nanomaterial life-cycle for the coating industry. The comparison of identified release scenarios (e.g. Brouwer 2010) with available release studies (e.g. Kuhlbusch et al. 2011) showed that until now only little attention was spent on the spray application of coatings.

In 2012, a total number of more than 5.5 billion spray cans were produced in Europe. Around 200 million cans of them were filled with coatings, whereof about 75 million were made in Germany (FEA European Aerosol Federation 2013). Losert et al. (2014) reviewed the few studies regarding the release of nano-objects from spray products, which were performed without exception by chamber and room experiments. For example, Hagendorfer et al. (2010) have analyzed the nanometre size range of spray aerosols that originated from aqueous silver-nanoparticle suspensions, which were aerosolized by propellant and pump spray cans within a test chamber. Aside from these, some published studies exist, which deal with the analysis of coating spray aerosols in the size range above 300 nm (e.g. Brosseau et al. 1992; Carlton and Flynn 1997; Sabty-Daily et al. 2005).

The aim of the present study was to fill this gap by granulometric characterization of coating spray aerosols originating from two types of commercial propellant spray cans and a manual gravity spray gun. In order to solve the complex metrological challenge, orientation measurements were performed firstly in an industrial spray booth. The gathered data regarding the magnitude of particle number concentrations and particle sizes will not discussed here but served for the design of a laboratory spray-channel and for a suitable experimental setup. In a second step, the spray processes were macroscopically characterized before the actual (nano)-particle release analyzes were performed. The (nano)-particle release characterization was carried out according to the approach used in Göhler et al. (2010) and which is described more detailed in Göhler et al. (2013).

## Materials and methods

### Spray application units

Three different coating spray-application technologies in the field of domestic use and handcraft were analyzed in this study, i.e. standard spray cans (SSC), SprayMax^®^-cans (SMC) and a manual gravity spray gun (SGA).

 SSCs, which based on propellant gas and circular-stream-atomization, are typically operated in domestic use. Spray cans based on the SprayMax^®^-technology (patent specification DE9636221 and US5957341) are used in the area of handcraft. They have a higher ejection mass flow than SSCs due to higher inner propellant pressure. Additionally, SMCs are typically operated with broad-stream atomization.

Pressurized air spray guns are more common used in the area of handcraft as SMCs. For the spray gun application (SGA), a high volume low pressure (HVLP) manual gravity spray gun (Model W300 08 G200, Anest Iwata Corporation, Japan) with a 0.8 mm nozzle was operated at 2.5 bar system pressure in circular-stream-mode.

## Materials

### Coatings and tracer nanoparticle additives (TNPA)

The four coating systems, which were analyzed in this study, are given in Table 1. The first two coating systems (PU, ACL) are typically used in the domestic field, whereas the last two ones (WL, LML) are applied in the area of handcraft and industry.

**Table 1:** Coating systems, analyzed spray processes and sample identification key

Coating system	TNPA	SSC	SMC	SGA	Sample ID
Two-pack polyurethane coating	–	x	x		PU
	ZnO^a^	x	x		PU–ZnO
	Fe_2_O_3_^b^	x	x		PU–Fe_2_O_3_
Acrylate topcoat with TiO_2_ pigment particles	–	x	x		ACL
	ZnO^a^	x	x		ACL–ZnO
	Fe_2_O_3_^b^	x	x		ACL–Fe_2_O_3_
Water-based coating with TiO_2_ pigment particles	–	x	x	x	WL
	SiO_2_^c^	x	x	x	WL–SiO_2_
Organic solvent-based mixed coating	–	x	x	x	LML
	SiO_2_^c^	x	x	x	LML–SiO_2_

^a^Formulation LP-X 21217

^b^Formulation JS-08-032A

^**c**^Hydrophobized synthetic amorphous silica

Three different kinds of metal-oxide tracer nanoparticle additives (TNPA) were deliberately admixed to the coating systems. The employed ZnO- and Fe_2_O_3_-TNPAs were the same ones as used in previous studies (Vorbau et al. 2009; Göhler et al. 2010). The ZnO-TNPA with a number weighted median diameter of *x*_50,0,ZnO_ = 75 nm and a nanoparticulate fraction of *Q*_0,ZnO_(100 nm) = 75 no% are finer but broader distributed than the Fe_2_O_3_-TNPA with a median diameter of $x_{50,0,{\text{Fe}}_{2}{\text{O}}_{3}} = 115\,{\text {nm}}$ and a nanoparticulate fraction of $Q_{0,{\text{Fe}}_{2}{\text{O}}_{3}}$ (100 nm) = 25 no%. The TNPA SiO_2_ is an hydrophobized synthetic amorphous silica (SAS) that consists of fractal aggregates of sintered primary particles with an average primary particle diameter of 7 nm. The BET surface area is specified by the manufacturer with 220 ± 25 m^2^ g^−1^.

### Chemical composition within the spray units

**Fig. 1:**
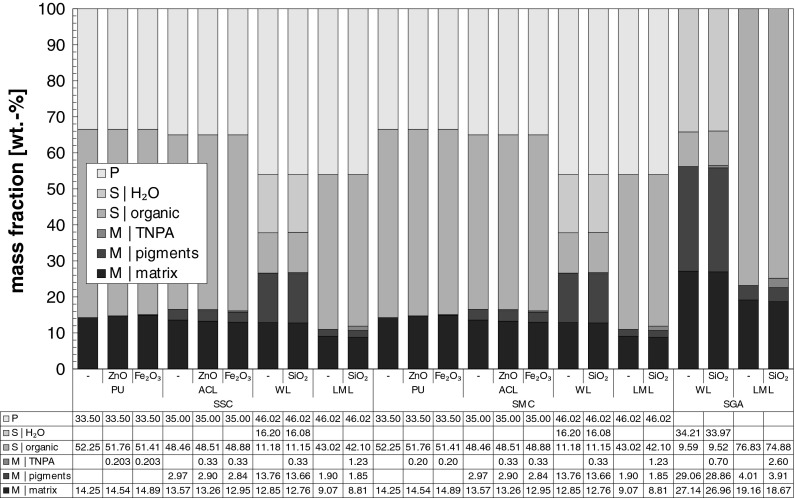
Chemical composition of the analyzed coatings as configured within the operated spray units

The rough chemical composition of the analyzed coatings within the different spray units is given in Fig. 1 and can be classified in three main categories, i.e. propellant (P), solvent (S) and solid matter (M).

The propellant-content of dimethyl ether (C_2_H_6_O) or of mixtures of butane (C_4_H_10_) and propane (C_3_H_8_) within the spray cans reached from 34 to 46 wt%. In contrast to the spray cans, the mass content of the spray gun propellant gas (particle free pressurized air) is not specified due to the possibility of different spray gun operating conditions.

The solvent-content comprises beside organic solvents and diluents also the content of water in the case of water-based coatings. Within the spray cans, the water-based coating solvent-content amounted 27 wt%, whereas the other coatings contained 42 wt% up to 52 wt% of solvent. Due to the lack of propellant specification, the solvent-content for the spray gun coatings is therefore higher and amounts to 44 wt% for the water-based coatings and to 75 wt% for the organic-solvent-based coatings.

The third category comprises the non-volatile components. Except the pigment-content and TNPA-content, all other solid components (e.g. binder, hardener, filler, dispersing agents) were summarized in the subcategory matrix. Considering the spray cans, the non-volatile content amounts 27 wt% for the water-based coatings and varied for the other coatings between 11 and 17 wt%. In the case of SGA, the solid content of the organic-solvent coatings amounts 56 and 25 wt% for the water-based coatings.

The TNPA content varies in the case of the spray cans (SSC, SMC) between 0.2 and 1.2 wt%, whereas it ranges from 0.7 to 2.6 wt% for SGA. Considering solely the non-volatile components (i.e. the final surface coating after application and drying), the content of the TNPAs ZnO and Fe_2_O_3_ would amount to values of 1.3–2.0 wt% and the SiO_2_-TNPA content would be 1.2 wt% for the WL-coating or 10.3 wt% for the LML-coating.

## Experimental details

### Spray-channel

**Fig. 2:**
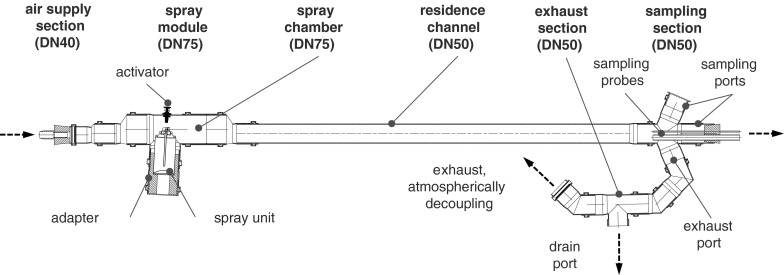
Sectional view of the developed spray-channel for aerosol characterization of spray cans and spray guns

Due to the multitude of aerosol-analytical disadvantages (e.g. relatively high setup times, fluidic dead zones, different residence times, poor mixing, concentration gradients) accompanied with the use of test chambers, a simple spray-channel (see Fig. 2) made from standardized polypropylene components (EN 1451-1:^1999^) was developed for the spray aerosol characterization. The main parts of the 1635 mm long spray-channel are an air supply section, a spray module with spray chamber for the inclusion of the spray units, a residence channel, a sampling and an exhaust section.

Within the spray-channel, the spray aerosol generated in the spray module is immediately gathered by the supplied particle free volumetric air flow and fed through the residence channel to the atmospheric decoupled exhaust access. The supplied turbulent volumetric air flow serves also for spray aerosol pre-drying, particulate pre-dilution, pre-reduction of volatile organic components (VOC, based on propellant and organic solvents) and for providing a homogeneously distributed particle concentration over the channel cross section. The distance between the nozzle outlet of the spray units and the entrance to the sampling tubes of the sampling section was set to 1,235 mm.

**Fig. 3:**
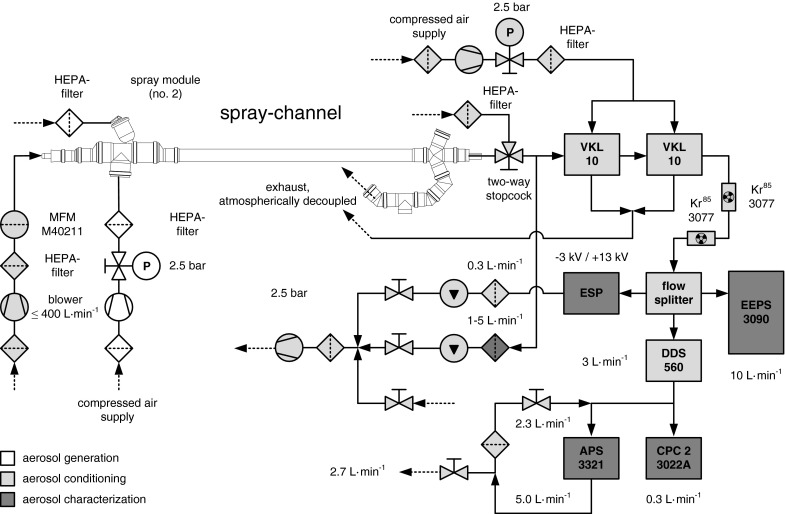
Schematic diagram of the experimental setup for spray application, aerosol conditioning and characterization

The spray module shown in Fig. 2 is the one used for the spray cans. Different adapter-designs allow the inclusion of various spray can geometries (e.g. nominal volume of 150, 250, 400 mL). In the case of SGA, a similar spray-module was used, wherein the spray gun was positioned and protruding components were thread through sealed hollows as shown in the schematic diagram of the experimental setup in Fig. 3.

### Instrumentation

The target granulometric measurands for the spray aerosol characterization down to a few nanometres are the number weighted particle size distribution (PSD_0_), the particle number concentration (PNC) and the elementary composition of the released particles. Currently, these measurands cannot be determined by a single measurement device. Therefore, different instruments were selected and combined in this study to obtain a general view of the particulate spray emissions.

An Engine Exhaust Particle Sizer (EEPS, Model 3090, TSI Inc., Shoreview, USA), a fast electrical mobility aerosol spectrometer (e.g. Biskos et al. 2005), was used for the temporally high-resoluted determination (10 Hz) of PSD_0_s from 5.6 nm up to 560 nm. The PSD_0_ of coarser spray aerosol particles in a range between 0.5–20 µm was measured by an Aerodynamic Particle Sizer (APS, Model 3321, TSI Inc., Shoreview, USA), a time-of-flight optical particle counter (e.g. Wilson and Liu 1980). EEPS and APS determine both PGV_0_ and PNC. For the purpose of redundancy verifying, a condensation particle counter (CPC, Model 3022A, TSI Inc., Shoreview, USA), which bases on magnifying aerosol particles to an optical-detectable size by heterogeneous condensation (e.g. McMurry 2000), was used for the highly-sensitive detection of PNCs in a size range from 6 to <10 µm. Imaging analyzes by scanning- (SEM) and transmission electron microscopy (TEM) and elementary analyses by energy dispersive X-ray spectroscopy (EDX) were performed on spray aerosol particles, which were deposited on substrates within an electrostatic precipitator (ESP; Dixkens and Fissan 1999).

In addition to the aerosol measurement instruments, different devices and procedures were necessary to achieve best possible measuring conditions. Thus, two different kinds of dilution systems were operated. A dynamic dilution system (Model DDS 560, ToPAS GmbH, Dresden, Germany) based on bypass-filtration was operated solely for a defined reduction of the PNC. External air dilution units (Model VKL 10, Palas GmbH, Karlsruhe, Germany) were used for a defined reduction of PNC and VOC (Koch et al. 1988; Helsper et al. 1990). For the purpose of aerosol neutralization, radioactive Kr^85^ bipolar neutralizers (Model 3077, TSI Inc., Shoreview, USA) were employed.

### Experimental setup

Figure 3 shows the schematic diagram of the experimental setup that was operated for the particle release characterization during SGA. In the case of the spray cans, the same experimental setup was used but except the components for spray gun operation. The spray-channel was continuously purged by a defined volumetric flow between 200–300 L min^−1^ of HEPA-filtered air for spray aerosol transportation, drying and pre-dilution of PNC and VOC.

The extraction of the aerosol sample flow was realized by one respectively two VKLs, which were operated with dry (<10 % RH) and HEPA-filtered compressed air at a system pressure of 2.5 bar. Beside a defined particulate reduction, these dilution units allowed also a concentration decrease of VOC that was essential for occupational and instrumental safety.

Before entering the flow splitter, the aerosol was passed through a cascade of two bipolar neutralizers. Both the EEPS and the ESP got their aerosol sample without further dilution procedures, whereas the sample flow for the CPC and the APS was fed firstly in a DDS. The sample flow of the APS was additionally diluted by partial backmixing with HEPA-filtered device exhaust.

For the operation of the experimental setup, non-conductive tubing was installed before the neutralizers, whereas conductive tubing was used after the neutralizers.

### Experimental procedure

The experimental procedure was carried out stepwise. Firstly, the spray units to be analyzed were activated, i.e. the mixture of the two-pack coatings were initiated for the spray cans respectively the gravity feed cup of the spray gun was filled. Afterward the spray units were gravimetrically analyzed using an analytical balance (Model BP310S, Sartorius AG, Göttingen, Germany). The spray cans (SSC, SMC) were then shaken manually not less than 30 s and further agitated by a laboratory shaker (Model IKA^®^ MS3 digital, IKA^®^-Werke GmbH & Co. KG, Staufen, Germany) for 60 s at an agitation stroke of 4.5 mm and a rotational frequency of 3,000 min^−1^.

**Fig. 4:**
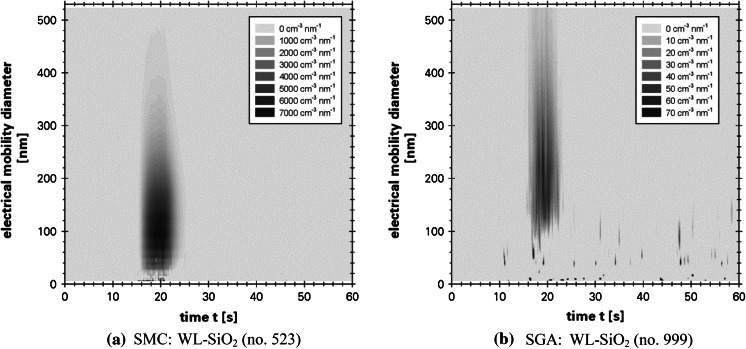
Visualization of the experimental procedure during EEPS data logging on the example of two single analyzes (q_0_·c_n_ ≡ dc_n_/dx); EEPS electrometer noise corrected

After assembling with the spray units, the spray-channel was firstly purged for 10 s with particle-free air before the actual data logging for 60 s began. The first 10 s of the measurement period were performed without any action to receive data for the offset-correction of the inherent EEPS electrometer noise. This was followed by the actual spraying process that lasted for 5 s. During the remaining measurement time, no further interventions were performed. The described measurement procedure led to a characteristic progression in the measurement data as exemplarily shown for the EEPS-data in Fig. 4.

Subsequently, the spray units were removed from the spray-channel and again gravimetrically analyzed. The whole experimental procedure was performed five times for each analyzed coating in the case of SGA. For the spray can application (SSC and SMC), a total of 6–12 single measurements were carried out using 2–4 single cans for each coating configuration.

After the completion of a measurement series for a coating configuration, the substrates for SEM-, TEM- and EDX-analyses were removed from the ESP and immediately stored in airtight sample containers, which were decontaminated afore within a laminar flow bench (Modell LF-VM-K0615; Steag Laminarflow Prozesstechnik GmbH, Pliezhausen, Germany) by purging with HEPA-filtered pressurized air. The substrate removal lasted approximately 3 s, where each substrate was exposed to the laboratory atmosphere. To minimize potential contaminations, the laminar flow bench next to the experimental setup was operated the whole time during the measurement campaign. Prior the next measurement series, the spray-channel and the complete tubing were purified in order to avoid cross contaminations.

## Results and discussion

### Macroscopic spray process characterization

In order to determine quantitative release data, macroscopic ejection parameters of the spray units were examined before, during and after the release analyzes.

**Fig. 5:**
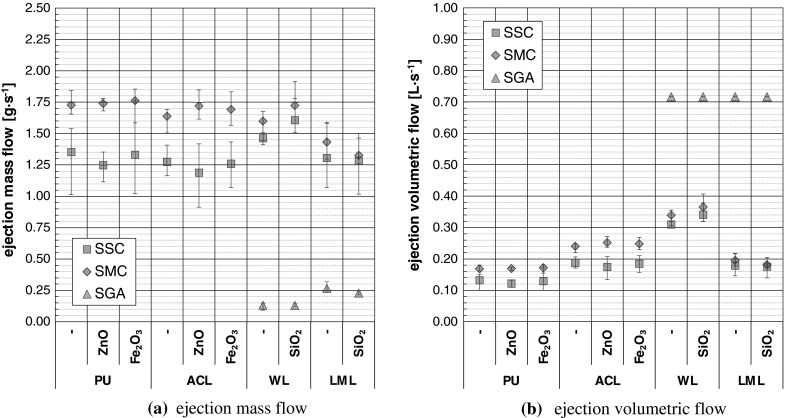
Ejection parameters of the spray cans (SSC, SMC) and the spray gun (SGA); *error bars* = data spreading of 5–12 single measurements

Figure 5a gives the ejection mass flows determined during the release analyses for a spray duration of 5 s. The SMCs showed the highest ejection mass flows over all analyzed coating configurations, whereas the lowest ones resulted for SGA.

The determination of the ejection volumetric flows of the spray cans shown in Fig. 5b based on gravimetrical identification of the displaced water mass that originated from the inflation of rubber bags. After correction by the bags surface tension and the acting water column, the ejection volume was related to simultaneous determined ejection masses. It was found that the ejection volume of the analyzed spray cans is directly proportional to the ejection mass irrespective of the spray can kind (i.e. SSC or SMC), the spray can dimension (i.e. 150, 250 or 400 mL) or the TNPA admixture. In contrast, it could be proved that the ratio of ejection volume and ejection mass depends on the containing coating system. The determined proportionality factors (*a*_PU_ = 97.5 mL g^−1^, *a*_ACL_ = 146.8 mL g^−1^, *a*_WL_ = 212.3 mL g^−1^, *a*_LML_ = 136.9 mL g^−1^) were used in context with the determined ejection mass flows shown in Fig. 5a and led to the ejection volumetric flows of the spray cans as given in Fig. 5b. The ejection volumetric flow of the spray gun was characterized (system pressure 2.5 bar, operation without coating) by means of a mass flow meter (Model 40211, TSI Inc., Shoreview, USA).

**Fig. 6:**
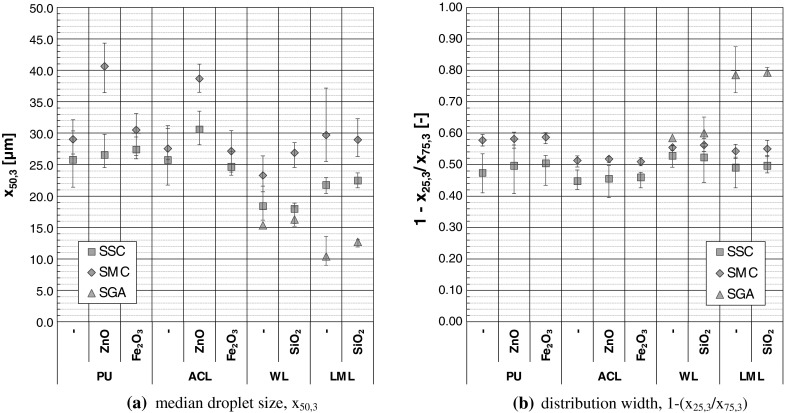
Characteristic parameters of the volume-weighted droplet size distribution (PSD_3_) at a distance of 10 mm from the nozzle exit, determined by laser diffraction analyzes; *error bars* = spreading of 15 repeated measurements

Beside the ejection mass flow and the ejection volumetric flow, the volume-weighted droplet size distributions (PSD_3_) of the spray units were determined in a distance of 10 mm to the spray nozzle outlet by means of a laser diffraction spectrometer (Model HELOS/KR-H2487, Sympatec GmbH, Clausthal-Zellerfeld, Germany) according to ISO 13320:^2009^. The HELOS was operated with a focal distance of *f*_R3_ = 100 mm to cover a size range between 0.9 µm up to 175 µm. The determined density functions of the PSD_3_ contained the isolated target spray modus/peak of around 25 µm and poorly-reproducible slopes in the density function at the upper and lower size range limits. The coarse droplets (*x* > 75 µm) have been visually observed before during the spray can analyzes, where they settled down in a distance between 10 and 50 mm from the spray nozzle exit. For the purpose of macroscopic droplet spray aerosol characterization, the target peaks were separated from the whole density function by a band-pass-filter algorithm. Afterward the characteristic parameters as shown in Fig. 6 of the target droplet peak were determined.

Regardless the kind of application unit, the volume-weighted median droplet diameter (x_50,3_) of the analyzed coatings reached from 10 to 45 µm as shown in Fig. 6a. The SPAs led to the finest (see Fig. 6 a) but broadest (see Fig. 6b) PSD_3_s with *x*_50,3_ = (10–20) µm. The coarsest droplet aerosols with *x*_50,3_ = (20–45) µm were determined for the SMCs, whereas the narrowest PSD_3_s were detected for the SSCs. Comparing solely the two different kinds of spray cans, the droplet aerosols of the SSCs were in most cases finer than for the SMCs.

No significant impact on the droplet spray-aerosol characteristics could be attributed to the TNPA-admixture with Fe_2_O_3_ and SiO_2_. The observable significantly higher *x*_50,3_-values of the ZnO-doped coatings in comparison to their non-doped counterparts during SMC-application based less on the ZnO admixture but rather on the whole SMC-confection. The macroscopic results for the SSC-application and also the above-mentioned finding for the TNPAs Fe_2_O_3_ and SiO_2_ confirm this conclusion.

## Spray aerosol characterization

### Number-weighted particle size distributions (PSD_0_)

**Fig. 7:**
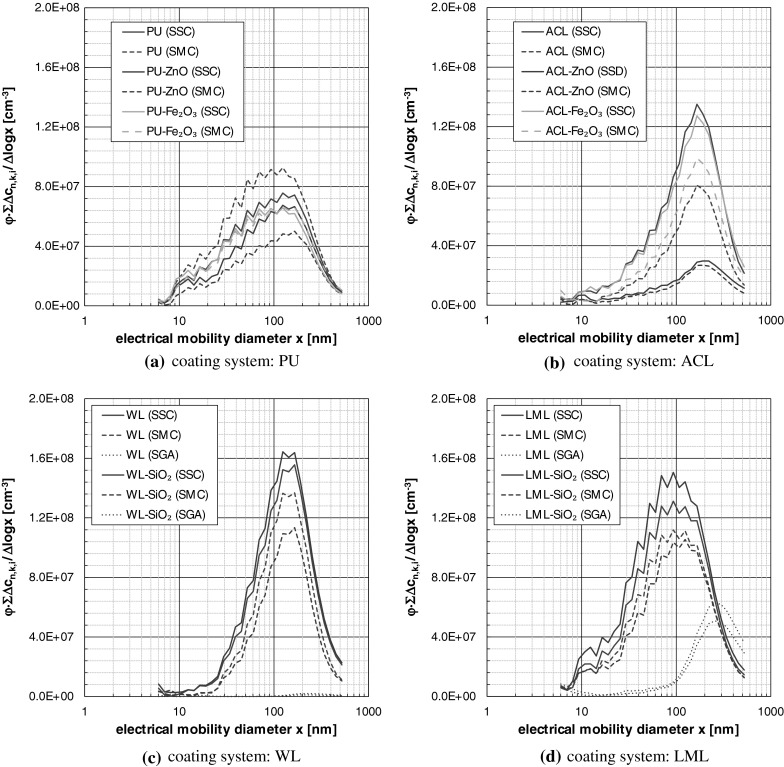
Transformed and scaled number weighted particle size distributions determined by EEPS; data based on the first of the 5–12 performed analyses for each configuration

To visualize the size distribution and the relative amount of released particles over the whole measurement time, the PNC of each class (class index *k*) was accumulated over the time increments (time index *i*) of the measurement procedure, corrected by the used dilution factor *φ* and related to the class width. This procedure was performed using the EEPS-data and logarithmic class width and led to the transformed and scaled number-weighted PSD_0_ of released particles as shown in Fig. 7.

The spray can aerosols (SSC, SMC) are very similar to one another for the particular coatings regarding the curve shape. In contrast, significant differences can be observed comparing the spray can aerosols and the spray gun aerosols, especially for the water-based coatings (see Fig. 4). Excepting the water-based coatings, all size distributions show a secondary peak around 10 nm and below, that could be originate from the formation of secondary organic aerosol (SOA) particles due to the high amount of VOC. The relaxation process could maybe caused a SOA formation. A formation initiated by the corona charger of the EEPS can be excluded, because despite the flow circuit within the EEPS no continuous SOA-peak could be observed.

Beside the curve shapes, the scaled size distributions of Fig. 7 allow also an estimation of the relative release amount under the premise of comparable process and analytical parameters. In contrast to the SGA, the ejection parameters (see Fig. 5) of SSC and SMC differ slightly and a first estimation is possible. The size distributions show that the SMC-application led in 9 of 10 cases to a minor release than the SSC-application. The only exception occurred for PU–ZnO. More detailed and robust findings are only possible taking into account the determined spray process parameters and analytical parameters for aerosol conditioning and sampling as shown in the next paragraph.

The PSD_0_s determined by the APS were very similar among one another and show only the leftover slope of the total PSD_0_ in direction to coarser particles. In this context, the representation in Fig. 10 should suffice here. In fact, Fig. 10 shows the transformed PSD_0_ (for a single analysis), which were correlated to the device-specific release numbers of EEPS and APS for the purpose of comparison. Despite the different equivalent diameters, it is evident that the APS supplemented well the EEPS size range. This based on the fact that the spray aerosols consist of spherical and compact particles with nearly unit-density. The decrease in the APS–PSD_0_ for *x* < 0.8 µm is not an evidence for a bimodal PSD as often misinterpreted in the literature, but a typical artifact of the measuring device that bases on the reduced values in the counting efficiency curve towards the lower detection range limit.

**Fig. 8:**
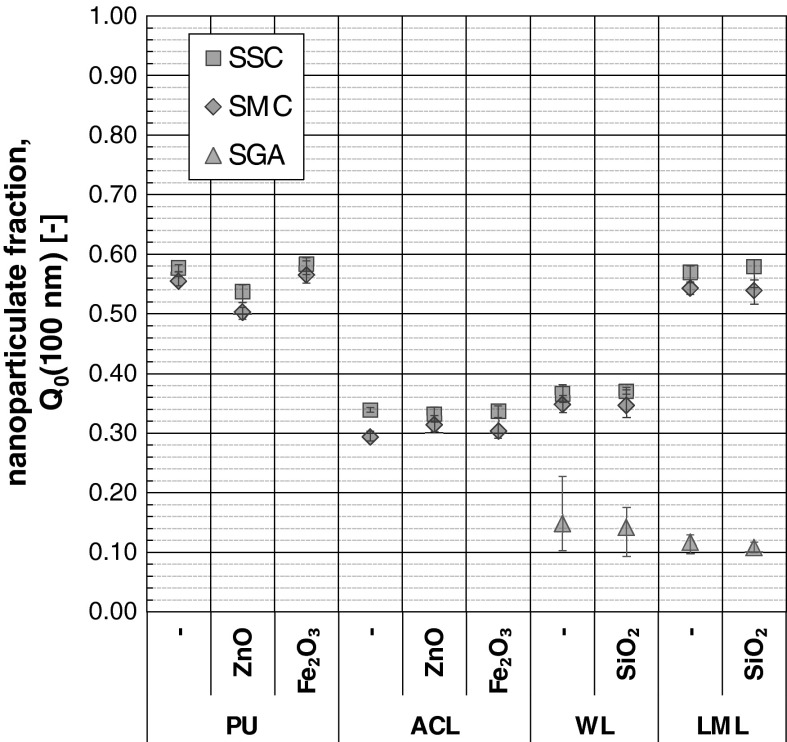
Nanoparticulate fraction Q_0_(100 nm) based on EEPS measurement data; *error bars* = data spreading of 5 (SGA) respectively 6 (SSC, SMC) repeated measurements

The nanoparticulate fraction Q_0_(100 nm) of the dried spray aerosols based on the EEPS measurement data are shown in Fig. 8. The specified values would be lower taking into account also the coarse fraction of *x* > 560 nm.

The nanoparticulate fraction of the analyzed coatings and spray units reaches from 10 to 60 no%. It is evident that the nanoparticulate release depends on the coating system and the application unit. The lowest nanoparticulate fractions arise for the SGA with values between 10 and 20 no%, whereas the highest ones yielded for the SSCs. The nanoparticulate fraction amounts from 30 to 60 no% for the application by SSC and SMC. The additivation of the coatings with the TNPAs Fe_2_O_3_ and SiO_2_ shows no systematic differences to the non-doped reference coatings in the nanoparticulate fraction. Compared with the coating PU, PU–ZnO shows a slight lower nanoparticulate fraction that is attributed to the spray can confection as discussed above.

### Specific fractional particle release numbers

**Fig. 9:**
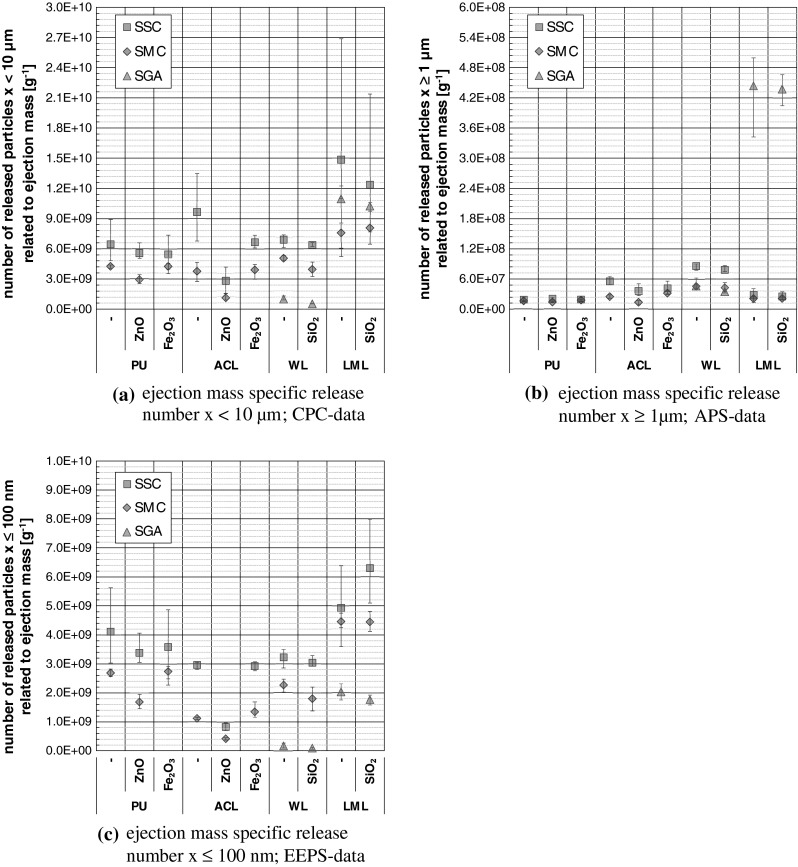
Ejection mass specific fractional numbers of released particles; *error bars* = data spreading of 5 (SGA) and 6–12 (SSC, SMC) repeated measurements

Based on the adjusted aerosol-analytical parameters (volumetric flow rates, dilution ratios) and the recorded PSD_0_s and PNCs (see Fig. 4), fractional release numbers were determined and related to the ejection mass of the spray units. For the purpose of comparison within this work or with other release studies, it should be noted that the ejection mass of the spray cans comprises in addition to the solid matter also the propellant and the solvent, whereas the ejection mass of the spray gun consists no propellant content. Using only the solid content, the values of the following release data would be 4–10 times higher as specified. Furthermore, due to congruent experimental procedures, it is possible to multiply the ejection mass flows given in Fig 5 a with the corresponding values of the specific release numbers of Fig. 9 to obtain the absolute particle flux, which is also known as particle release rate.

Annotations on the evaluated fractions of released particles are given in Table 2, whereas further information can be drawn from the remarks in Göhler et al. (2013).

**Table 2:** Annotations on the evaluated fractions of released particles

Release fraction	Device	Annotation
*x* < 10 µm	CPC	Total particle release (6 nm ≤ *x* < 10 µm), most robust results, data based on measured concentrations below photometric mode of the device
*x* ≥ 1 µm	APS	Number of released particles in the micrometre size range (1 µm ≤ *x* ≤ 20 µm); data based on aerodynamic particle diameter without Stokes correction
*x* ≤ 100 nm	EEPS	Number of released nanoparticles (5.6 nm ≤ *x* ≤ 100 nm), data based on electrical mobility particle diameter

Figure 9 shows the determined ejection-mass specific fractional numbers of released particles. It is evident that the particle release numbers depend on the operated spray unit and the used coating system. Regardless the kind of coating and spray unit, the total particle release numbers reach from 5.2 × 10^8^ to 2.7 × 10^10^ g^−1^, whereas the micro-particle release numbers lie between 8.7 × 10^6^ and 5.0 × 10^8^ g^−1^ and the nanoparticle release numbers extend over 5.3 × 10^7^ × 8.0 × 10^9^ g^−1^.

Considering only the spray can operation, the SSCs led for all size fractions to higher particle release numbers than the SMCs. The admixture with the TNPAs Fe_2_O_3_ and SiO_2_ shows no systematic impact on the particle release numbers. The ZnO doped coatings show partly less particle release numbers as their non-doped counterparts that is attributed to the spray can confection as discussed above.

### SEM, TEM and EDX

Extensive SEM-, TEM- and EDX-analyses on electrostatically deposited spray aerosol particles were performed and led to detailed informations on the species of the dried spray aerosol particles. In the case of dried droplets finer than 200 nm, the matrix sheath became more and more diffuse in the SEM-images, so that the thin matrix sheath around single pigments and also the fine pure matrix material droplets itself were not visible during the SEM-analyses (Model SEM, Model Gemini 982, Karl Zeiss AG, Jena, Germany) at 3 kV acceleration voltage, but could be proofed by the performed TEM-analyses (Model Tecnai 20, FEI Company, Hillsboro, USA).

**Fig. 10:**
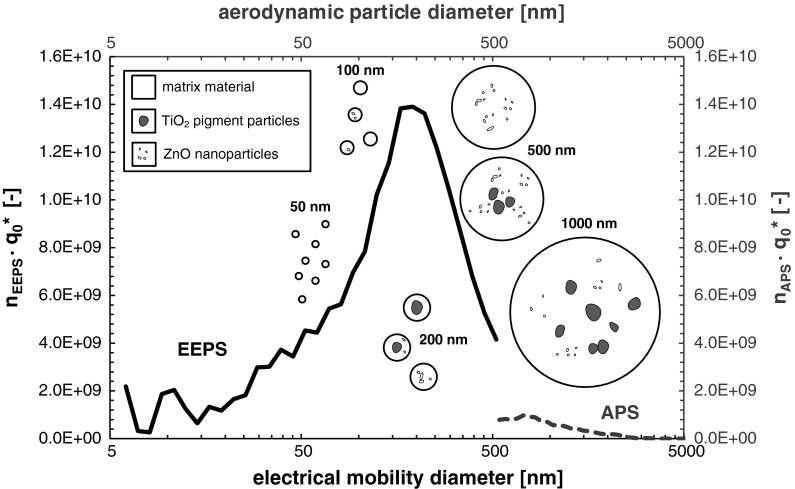
Transformed PSD_0_ correlated to the device-specific release number of white-pigmented acrylate coating with ZnO-TNPAs during SMC-application (single analysis no. 455) with schematic illustration of the different kinds of dried spray aerosol droplets observed during SEM- and TEM-analyses

The schematic illustration in Fig. 10 visualizes the kind of particles recovered for the coating systems containing both TiO_2_ pigment particles and TNPAs. The coarse particle fraction extends from around 5.0 µm down to 0.5 µm, where the dried matrix droplets showed both embedded pigments and TNPAs. The middle particle fraction reaches from around 500 nm down to 100 nm. Within this fraction, dried matrix droplets with both pigments and TNPAs, dried matrix droplets either with embedded pigments or with TNPAs and pure matrix particles were identified. The fine particle fraction consisted solely of dried droplets made from matrix material without any pigment particles or TNPAs.

Beside the typical spherical dried droplets, also some large edged particles and a few Si-containing fractal agglomerates without matrix-sheath were observed. The former ones are attributed to fragments of formed thin coating films that were re-entrained from the spray nozzle exits, especially from the SGA. The fractal agglomerates were recovered for nearly all analyzed coating systems, e.g. also for those without deliberately admitted SiO_2_-TNPAs. The agglomerates showed furthermore a deviant structure in comparison to typically SiO_2_-TNPA agglomerates. Therefore, it is believed that the observed Si-containing fractal agglomerates may originated rather from incompletely dispersed clusters of other additives or extenders, which were forced open during the spray processes. The true origin of the fractal Si-containing agglomerates could not be fully elucidated within this study. Furthermore, no isolated ZnO- and Fe_2_O_3_-TNPAs were observed during the SEM- and the TEM/EDX-analyses.

**Fig. 11:**
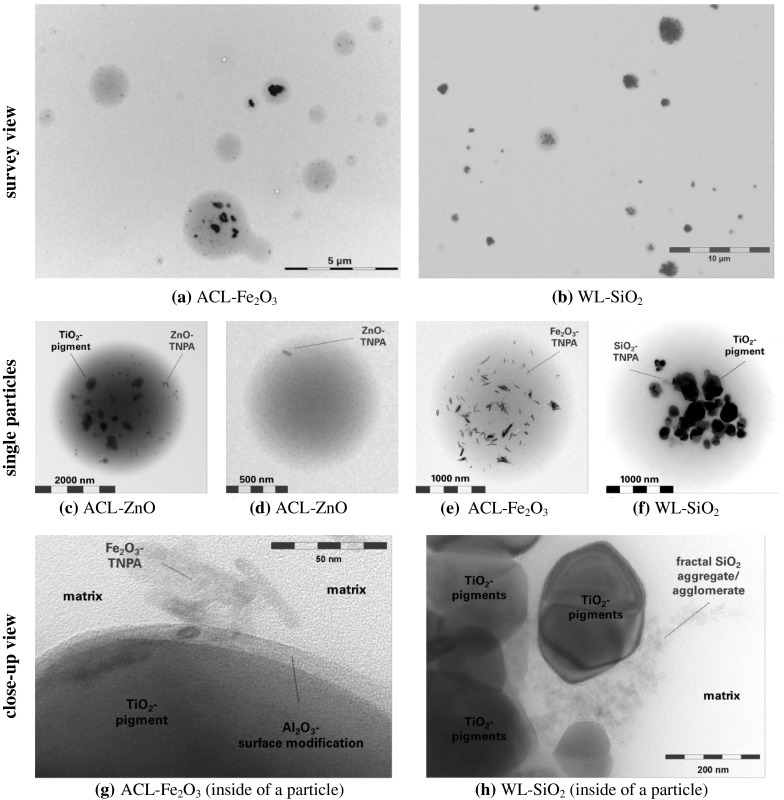
TEM-images of electrostatically deposited spray aerosol particles

Characteristic TEM-images of electrostatically deposited spray aerosol particles are shown in Fig. 11. It is evident that the used TNPAs ZnO and Fe_2_O_3_ in combination with the manner of the performed precipitation (substrate: carbon-coated TEM grids made of copper, Modell SF162, Plano GmbH, Wetzlar, Germany) are well-suited for their recovery even at lower magnification, whereas the used TNPA SiO_2_ is less suitable because of the lower material contrast and the concomitance of other Si-containing additives that were typically processed within coatings.

## Summary and conclusion

The airborne (nano)-particle release during spray application of nanoparticle-doped coatings by two kinds of propellant spray cans and a manual gravity spray gun was granulometrically analyzed. Therefore four types of coatings were doped with three kinds of metal-oxide TNPA (ZnO, Fe_2_O_3_, SiO_2_). The spray application occurred in a spray-channel, which was integrated in an experimental setup for qualitative and quantitative analyses on dried spray aerosols.

The granulometric results have shown that the spray application led to a particulate release in order of 5 × 10^8^ to 3 × 10^10^ particles per gram ejection mass, whereof between 10 no% up to 60 no% were finer than 100 nm. The represented release data based on worst-case conditions. On the one hand, the total spray jet without obstacle was captured for the analyses, i.e. no plane surface was coated. Thus, the simulated application process is more comparable with spray-coating of e.g. wire-mesh fences or bicycle baskets. On the other hand, optimal measurement conditions were realized for the analyses by preventing particle losses and particle coagulation to freeze the aerosol-condition immediately after the spray-nozzle exit.

To evaluate the more common used PNC from determined release data, defined model rooms were introduced in previous studies (e.g. Göhler et al. 2010, 2013, 2014). In the case of spray application, it is obviously to use the ejected propellant gas volumetric flow (Q_PG_ ≈ 0.1–0.7 L s^−1^). Doing this, the PNC within the propellant gas cloud would amount values between 5 × 10^5^and 3 × 10^7^ cm^−3^. Similar concentrations were measured for example in 2 m distance away from a typical cooking emission source (gas stove) due to grilling of 100 g bacon for 10 min in laboratory with a room volume of 50 m^3^ by Manigrasso et al. (2013). However, in the dependence of local airflows, the PNC of the spray clouds would reduce by mixing with air and can thus vary over magnitudes in practice. For example, the spray application of the analyzed acrylate coating (number of released particles *x* < 10 µm related to ejection mass ≈ 1 × 10^10^ g^−1^; ejection mass flow 1.25 g s^−1^) by means of a standard spray can for a duration of 10 s in a room with a volume of 10 m^3^ would lead under the premise of ideal mixing/dispersing conditions to a PNC of 1.25 × 10^4^ cm^−3^. This value lies within the same order of magnitude as e.g. urban particle number concentrations in offices (e.g. Lonati et al. 2010).

The number weighted PSD_0_ logged by EEPS showed beside the central peak around 100 nm also a fine fraction around 10 nm and below, that could maybe addressed to SOA formation.

Comparing the nanoparticle-doped coatings with the non-doped ones, the admixture of 0.2 wt% up to 2.6 wt% TNPA showed no tracer nanoparticle attributed impact on both the macroscopic spray process characteristics and the particle release numbers.

The SEM- and TEM-analyses proved particles made up solely from matrix material and matrix-sheathed pigments, fillers and TNPAs. Isolated ZnO- or Fe_2_O_3_-TNPA could not be observed. Nevertheless, imaging analyses suffer from comparatively low number of analyzed particles. Thus, statistically reliable particle material identification measurement methods are one aim for future work in this field of research.

Based on the findings of this and previous studies, we believe that the quality of the ENO-admixture (dispersing state, surface wetting, surface modification) is an more important fact as considered so far, when discussing about potential ENO release.
